# Extract of a Letter from Dr. Peebles

**Published:** 1849-01

**Authors:** 


*Extract of a Letter from Dr. Peebles,*
*Lexington, Mo., to Cin. Ed.:—*

A most remarkable case of Dental nudity came under my observation in Carroll county, Mo. Mrs. K. aged about 25 years, showed me her teeth, stating at the time, that "herself and father had teeth different from any body else." I found 32 sound teeth, entirely nude—not a particle of enamel on any one of them. They were very regular, not large, and worn to an even surface, and presented to the eye the appearance of healthy Dentine as seen in a sound fang in a healthy gum—an instrument cut them easily; she masticated all kinds of food with impunity. Not being prepared then, I failed to procure an impression, and she soon after removed West of the Rocky Mountains. H. E. P.

